# Investigation and analysis on the charging status and standard of pharmacy intravenous admixture service in China

**DOI:** 10.3389/fphar.2025.1648571

**Published:** 2025-10-21

**Authors:** Jie Cao, Zhou Shu, Jie Li, Dan Wang, Yong-Ning Lyu, Xue-Feng Cai

**Affiliations:** Department of Pharmacy, Union Hospital, Tongji Medical College, Huazhong University of Science and Technology, Wuhan, China

**Keywords:** pharmacy intravenous admixture service, current charging policy, charging standard, charging status, China

## Abstract

**Objectives:**

The charging policy of pharmacy intravenous admixture service (PIVAS) is not merely a minor “billing issue” confined to individual hospitals, but rather a critical component that impacts the safety, efficiency, equity, and long-term sustainability of the national healthcare system. Its implications extend across patients, medical institutions, the pharmaceutical industry, and even the broader public health landscape. To establish a foundation for the national charging standard of intravenous admixture service, this study comprehensively investigated and analyzed the operational costs and current charging policies of PIVAS across 30 provinces in China.

**Methods:**

Questionnaires were distributed through the “Wenjuan Xing” platform from May 6th to 1 July 2022. After data generation and export by the platform, statistical and descriptive analyses of the questionnaire results were conducted using statistical software, including EXCEL, SPSS.

**Results:**

A total of 761 PIVAS surveyed, 91.59% were affiliated with the pharmacy department, while 6.70% belonged to the hospital independent department. Most PIVAS strongly agreed with imposing fees, while most tertiary hospitals agreed that dispensing fees should be categorized based on hospital levels; however, most secondary hospitals disagreed. Approximately 60.58% of PIVAS have implemented a charging system which allows charges after inspection and evaluation. Regarding changes for different drugs nationwide, common drugs had an average charge standard of 4.39 yuan per bag while antibacterial drugs averaged 5.01 yuan per bag. Hazardous drugs had an average charge of 23.17 yuan per bag, whereas parenteral nutrition solutions averaged 38.75 yuan per bag. The annual operating cost of PIVAS in China was approximately RMB 2,098,100, with the integrated operating cost comprising 89.36% of the total, while dispensing cost accounted for only 10.64%. Human costs emerged as the highest annual consumption (74.20%), followed by facility maintenance (4.77%) and equipment acquisition costs (3.44%).

**Conclusion:**

The lack of a unified inspection and evaluation standard as well as charging standard in China is currently an urgent issue that needs to be addressed. The existing charging standard falls below the recommended level, thus it is necessary to develop a more reasonable and equitable charging standard that takes into account operational costs. This study can serve as an empirical reference for national medical insurance and health administration authorities in establishing unified regulatory standards and dynamic pricing adjustment mechanisms for PIVAS. It contributes to the transition of PIVAS from “regionally fragmented management” to “nationally standardized operations,” thereby supporting the dual objectives of enhancing healthcare service quality and optimizing the utilization efficiency of medical insurance funds.

## 1 Introduction

The Pharmacy Intravenous Admixture Service (PIVAS) is a department within medical institutions that offers professional and technical services for the centralized admixture of intravenous medications for patients. The establishment of PIVAS not only enhances the quality of finished infusion products, but also promotes rational drug utilization, optimizes human resource allocation, improves nursing work quality, and prevents occupational exposure among medical staff. Consequently, it has become an inevitable developmental trend in China’s hospital infusion drug dispensing (Hi, 2010). In December 2021, the Guidelines for the Construction and Management of Pharmacy Intravenous Admixture Service (Trial) issued by the National Health Commission have further advanced and reinforced the construction and management of PIVAS in medical institutions. Currently, over 2,000 medical institutions in China have established PIVAS, catering to 3–4 million beds (Yu and Wu, 2021), indicating a positive overall development.

The construction and operation costs of PIVAS are significantly higher than those of traditional pharmacies due to its distinct working model from the traditional pharmaceutical service model of pharmacy. Additionally, it also requires pharmacists to possess higher levels of professional and technical abilities. In 2004, Cao et al. (2004) conducted a comprehensive investigation and analysis of 11 hospitals in Shanghai that implemented PIVAS, revealing a significant “inverted” input-output pattern where only parenteral nutrition solutions were being charged.

The Zero-Markup Drug Policy necessitates a comprehensive assessment of the operational costs and benefits of PIVAS, as well as the establishment of reasonable charging standards. These factors serve as crucial foundations for ensuring the stable functioning and sustainable development of PIVAS (Pang et al., 2020; Tang et al., 2018; Xu et al., 2020; Wang and Xiang, 2016). However, currently in China, there is a lack of a standardized method for calculating and referencing PIVAS costs, resulting in a dearth of accurate regional benchmarks. Consequently, the formulated charge standards exhibit significant variations that fail to accurately reflect the service value provided by PIVAS pharmacists.

From May to July 2022, the National Institute of Hospital Administration under the National Health Commission of the People’s Republic of China and the Central Intravenous Admixture Service Committee affiliated with the Chinese Pharmacists Association jointly organized a nationwide research project on PIVAS for medical institutions. The survey covered PIVAS in medical institutions across 30 provinces, autonomous regions, and municipalities directly under the Central government (excluding Tibet Autonomous Region).

PIVAS currently faces three major challenges. First, the operational costs of PIVAS—including human resources, consumables, and equipment maintenance—have been increasing annually. However, the charging policy adjustment cycle is long and the process is complex, resulting in a situation where cost increases outpace fee increases, thereby continuously reducing cost coverage. Second, at the service level, efforts to control costs and reduce manpower may lead to an increased risk of service quality degradation. Third, at the development level, the imbalance between income and expenditure prevents most hospitals from investing in technological upgrades, leading to stagnant service efficiency and significant regional disparities (Yang et al., 2020).

With the continuous advancement of PIVAS and the emergence of intelligent technologies, intelligent supervision systems for drug dispensing based on image recognition, as well as integrated information platforms such as the “intelligent prescription review system combined with IoT-based dispensing equipment and an electronic traceability platform” (Deng et al., 2024; Wang et al., 2022; Tian et al., 2024), have become key focal points in recent years. These innovations are also essential for enhancing quality control, improving efficiency, and preventing risks (Qiu et al., 2024; Yin et al., 2023; Wang et al., 2025).

Therefore, establishing reasonable and fair charging standards is crucial to balancing PIVAS income and expenditure and promoting its sustainable development. This paper aims to comprehensively grasp the operational status, cost structure, current pricing practices, and existing challenges faced by PIVAS in China, thereby offering valuable insights for promoting high-quality and sustainable development of centralized intravenous admixture service.

## 2 Materials and methods

### 2.1 Participants

The survey subjects were selected from PIVAS in medical institutions across 30 provinces, autonomous regions, and municipalities directly under the Central Government (excluding Tibet Autonomous Region).

The survey period spanned from May 6 to 1 July 2022. Electronic questionnaires were disseminated via the “Questionnaire Star” platform, and within the research group, experts representing provinces, autonomous regions, and municipalities directly under the Central government took charge of coordinating with PIVAS leaders from relevant medical institutions in their respective areas to complete the questionnaires.

### 2.2 Survey content

The questionnaire utilized in this study was a self-designed non-scale instrument. The final version of the questionnaire was developed through discussions and consensus reached by the Institute of Hospital Management of the National Health Commission and the Central Intravenous Admixture Service Committee of Chinese Pharmacists Association.

The questionnaire encompassed seven sections, including: (1) hospital basic information; (2) PIVAS basic information; (3) PIVAS personnel situation; (4) PIVAS quality control; (5) current charging policy of PIVAS; (6) operating cost analysis of PIVAS; and (7) suggestions for PIVAS fees.

In total, the questionnaire consisted of 106 questions with an additional 241 sub-questions. For data analysis purposes, this paper focuses on examining responses to questions 63–106 related to “current fee policy,” “operating cost,” and “fee suggestion” within the overall questionnaire.

### 2.3 Statistical methods

The data statistics and descriptive analysis of the questionnaire results were conducted using Excel 19.0, SPSS 22.0, and other statistical software.

## 3 Results

### 3.1 Basic information of participants

A total of 722 hospitals responded to the questionnaire, and 761 questionnaires were collected (i.e., 761 PIVAS participated in the survey, with 27 medical institutions having two or more PIVAS).

**FIGURE 1:**
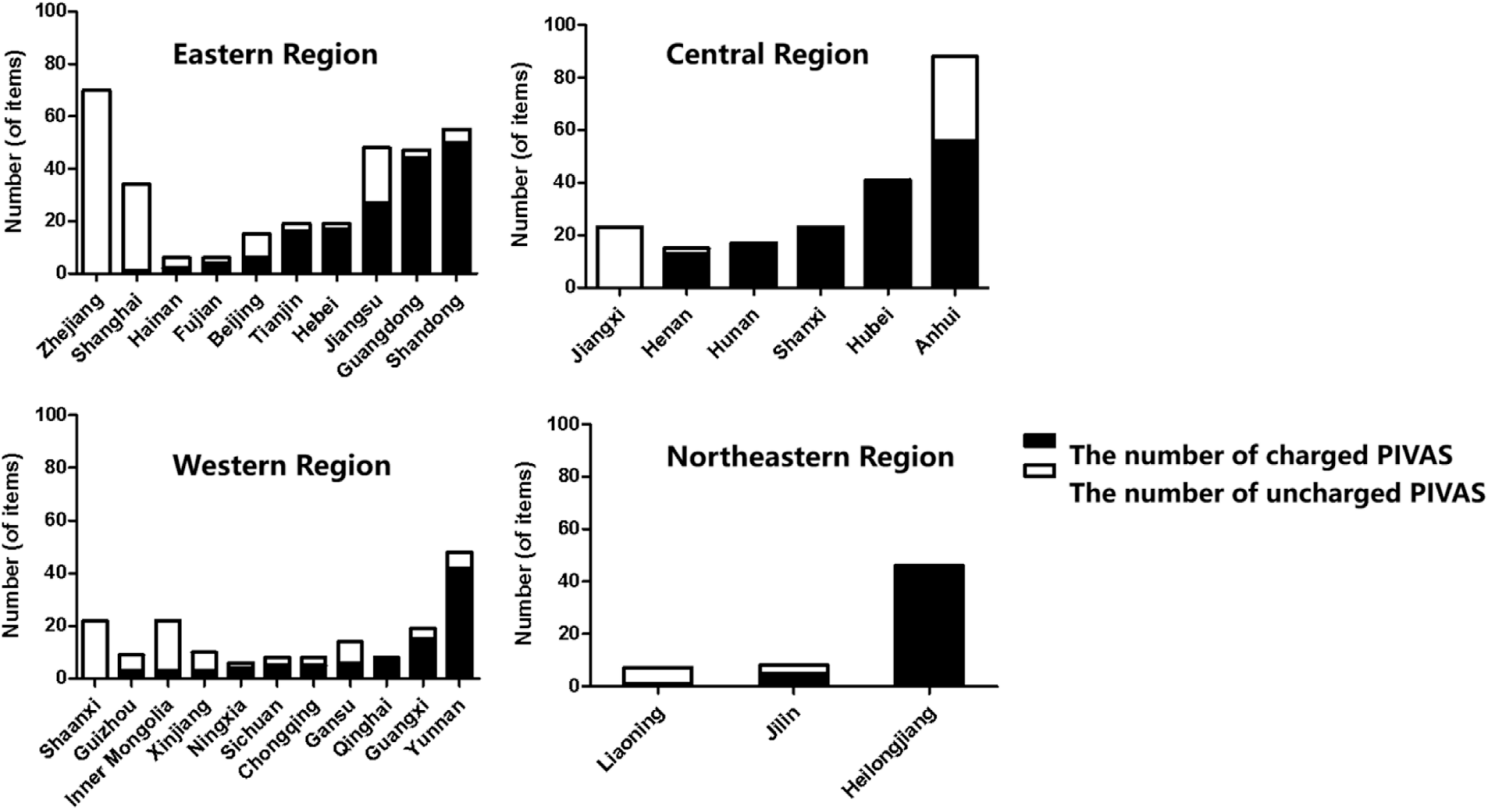
The numbers of fee-charging and non-fee-charging PIVAS in different regions.

Among the 761 PIVAS that took part in the survey, there were 174 PIVAS belonging to the western region, out of which 94 charged fees (54.02%). In the eastern region, there were a total of 319 PIVAS, among which 167 charged fees (52.35%). The central region had a count of 207 PIVAS, with a chargeable rate of 71.50% (148 chargeable). Additionally, there were also 61 PIVAS in the Northeast region, out of which a majority of them (52) were chargeable at a rate as high as 85.25%. The numbers of fee-charging and non-fee-charging PIVAS in each province, autonomous region, and municipality are presented in Figure 1.

The distribution of samples in this study is relatively concentrated in the eastern provinces, with fewer samples from the central and western regions, and a particularly limited sample size in the northeastern region. This uneven distribution may compromise the representativeness of the findings. Additionally, the questionnaire employed in this study was self-developed and lacks an established measurement scale, which may pose certain limitations regarding scale validity and related psychometric properties. Nevertheless, given the study’s extensive coverage, substantial sample size, and authoritative data sources, it represents one of the most comprehensive investigations to date and provides a reliable reflection of the current development status of PIVAS in China.

The survey findings indicate that PIVAS fees are imposed by medical institutions in Heilongjiang, Hunan, and Qinghai, whereas those in Jiangxi, Shaanxi, and Zhejiang do not levy such charges. In the remaining 24 provincial-level administrative regions, both charged and uncharged PIVAS coexist within the same jurisdiction. Possible reasons for this disparity include: (1) Some PIVAS facilities are still under construction or undergoing renovation without being fully operational; (2) Certain PIVAS units are currently undergoing acceptance evaluation and have not yet received approval to commence charging; (3) Charging policies vary among different cities and provinces within the same administrative region.

### 3.2 Investigation of the charging status of PIVAS in China

#### 3.2.1 The superior department of PIVAS

The survey results revealed that out of the 761 PIVAS, 91.59% were affiliated with the pharmacy department, while 6.70% belonged to the hospital independent department. Additionally, 0.66% were associated with the nursing department, 0.53% with pharmacy co-management, and 0.52% with either the preparation center, clinical pharmacy or under construction facilities. The detailed breakdown can be found in Table 1.

**TABLE 1:** The superior department of PIVAS in different regions.

Superior department	Total	East	Northeast	West	Central
Pharmacy department	697	304	47	152	194
Independent department	51	9	13	20	9
Nursing department	5	4	1		
Other (Nursing Pharmacy co-management)	4	2			2
Other (Preparation Center)	2			2	
Other (Clinical Medicine)	1				1
Others (to be built)	1				1

As indicated in Table 1, the majority of PIVAS are administered by the Department of Pharmacy, which can be attributed to their expertise in drug administration, dispensing, and supply. It is also plausible for certain hospitals to establish independent PIVAS departments based on their management structure and operational requirements. Furthermore, due to the coexistence of pharmacists and nurses, a hierarchical relationship with the nursing department and a collaborative approach towards drug and nursing management have emerged as corresponding modes of operation. Additionally, two PIVAS units fall under the purview of the pharmaceutical preparation center, which predominantly belongs to the Department of Pharmacy; this joint management arrangement with the pharmaceutical preparation center presents us with novel perspectives.

#### 3.2.2 Charge basis

The current charging basis of PIVAS in China is derived from the relevant documents issued by the Price Bureau of the provincial or municipal Development and Reform Commission, the Human Resources and Social Security Department (Bureau), the Medical Insurance Bureau, and the Health and Family Planning Commission (Health Commission) of each medical institution. As depicted in Table 1, out of 461 charged PIVAS, 85 PIVAS (accounting for 18.44%) indicated that PIVAS does not require inspection and evaluation for charging dispensing fees, while the remaining 376 pivas revealed that they do need to undergo inspection and evaluation. The organizations responsible for inspection and evaluation are primarily composed of the price bureau of provincial or municipal development and reform commissions, human resources and social security departments (bureaus), medical insurance bureaus, health commissions, drug management quality control centers, as well as other institutions which align with issuing units according to official documents. The standards for evaluation and inspection encompass acceptance criteria formulated by provincial or municipal business authorities for intravenous drug dispensing centers; regulations governing reviews on intravenous drug dispensing center practices; quality management standards for centralized intravenous drug dispensing; technical specifications regarding clean surgery department construction within hospitals; quality management measures pertaining to centralized intravenous drug dispensing; along with related charging documents.

#### 3.2.3 Charging standard

A statistical analysis of the questionnaires from 461 operational PIVAS facilities indicated that charges are imposed in 27 provincial administrative regions. Specifically, in Beijing, Gansu, Hainan, and Inner Mongolia, fees are not levied for common drugs or antibacterial drugs but only for hazardous drugs and parenteral nutrition solutions; while in Shanghai and Liaoning, charges are collected exclusively for hazardous drugs.

**FIGURE 2:**
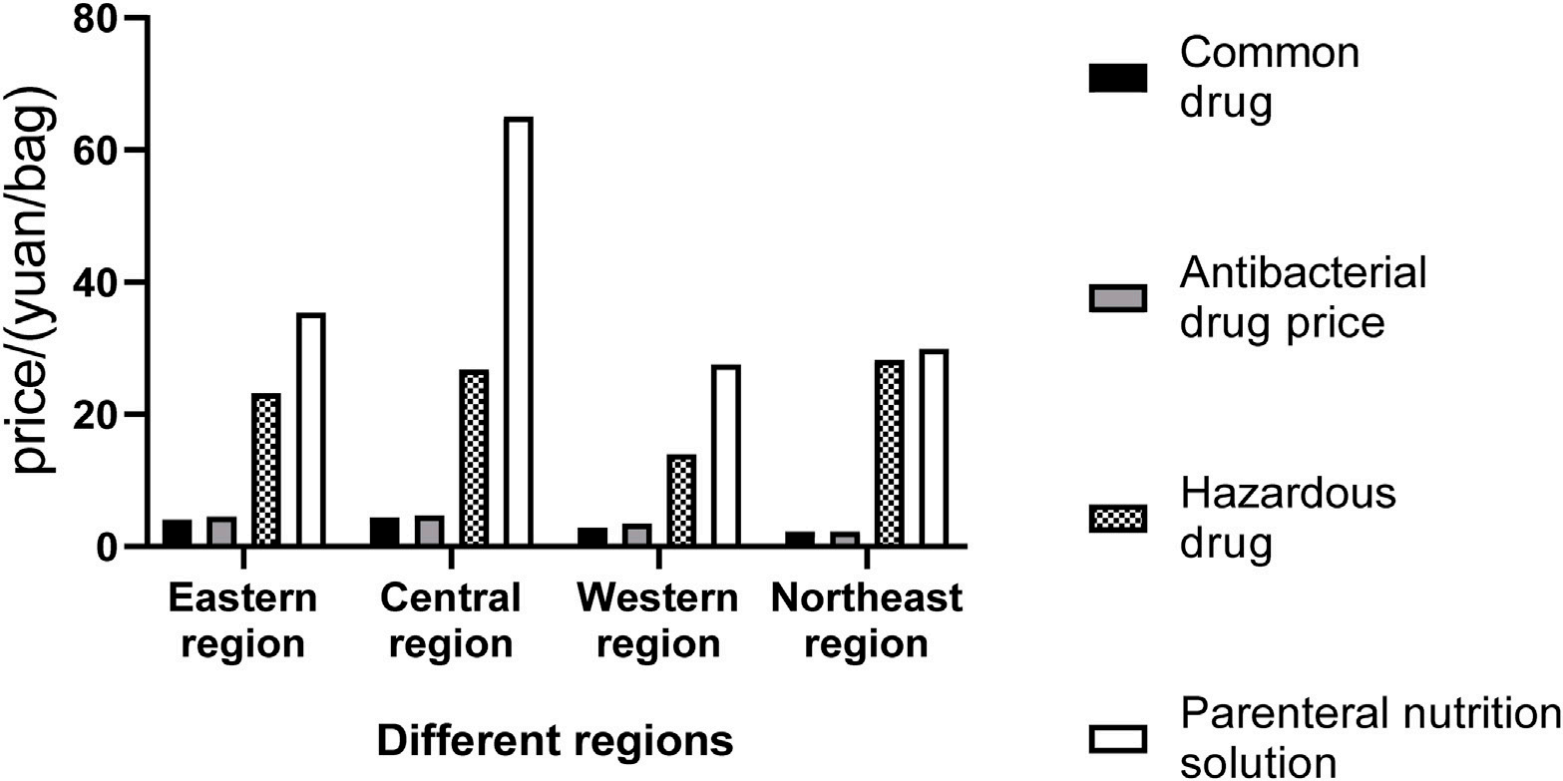
The average prices of different types of drug dispensing charges in PIVAS of different regions.

The average prices of different types of drug dispensing charges in PIVAS of each provincial administrative region are presented in Tables 2–5 and Figure 2. In conjunction with the relevant documents issued by the price authorities that impose PIVAS allocation fees in the corresponding regions, it can be observed that.

1. The national average charge standard for the dispensing of common drugs is 4.39 yuan per bag. The highest charge standard is in Jiangsu Province (9.32 yuan per bag). In Jiangsu, the government-guided price for the dispensing of common drugs is 9.90 yuan per bag (for tertiary public medical institutions). Secondly, there is Hubei Province (9.00 yuan per bag). In Hubei, the government-guided price for the dispensing of common drugs is 9.00 yuan per bag (for tertiary public medical institutions). The charging standards in Jilin and Fujian are the lowest, both at 1.00 yuan per bag.
2. The national average charge standard for the dispensing of antibacterial drugs is 5.01 yuan per bag. The highest charge standard is in Hubei Province, reaching 9.98 yuan per bag. In Hubei, the government-guided price for the dispensing of antibacterial drugs in tertiary public medical institutions is 10.00 yuan per bag. Next is Jiangsu with 9.40 yuan per bag. In Jiangsu, the government-guided price for the blending of antibacterial drugs in tertiary public medical institutions is 9.90 yuan per bag. The lowest charging standards are in Jilin and Fujian, both at 1.00 yuan per bag.
3. The national average charging standard for the dispensing of hazardous drugs amounts to 23.17 yuan per bag. The highest charging standard is observed in Hubei Province, attaining 52.44 yuan per bag. In Hubei, the government-guided price for the dispensing of hazardous drugs is 54.00 yuan per bag for tertiary public medical institutions. Secondly, it is Liaoning with 44.00 yuan per bag. The lowest charging standard is in Chongqing, standing at 5.70 yuan per bag.
4. The national average charging standard for the preparation of parenteral nutrition solution is 38.75 yuan per bag. The highest charging standard is seen in Hubei Province, attaining 90.68 yuan per bag. In Hubei, the government-guided price for the preparation of parenteral nutrition solution is 93.00 yuan per bag (for tertiary public medical institutions). Next is Henan (81.86 yuan per bag). In Henan, the government-guided price for the preparation of parenteral nutrition solution is 90.00 yuan per bag (in tertiary public hospitals). The charges in Chongqing and Sichuan are relatively low, which are 11.42 yuan and 9.40 yuan per bag respectively.

**TABLE 2:** The average prices of different types of drug dispensing charges in PIVAS of Eastern region.

Regions	Provinces	PIVAS quantity/pieces	Common drug price/(yuan/bag)	Antibacterial drug price/(yuan/bag)	Hazardous drug price/(yuan/bag)	Parenteral nutrition solution price/(yuan/bag)
Eastern region	Shandong	50	4.57	6.12	28.47	20.01
	Guangdong	44	4.39	4.56	20.26	18.82
	Jiangsu	27	9.32	9.4	38.13	40.64
	Hebei	17	3	4	7.79	63
	Tianjin	16	2	2	11	14.93
	Beijing	6			28	28
	Fujian	4	1	1	26.5	27.25
	Hainan*	2			22、39.28	70.24
	Shanghai	1			10	
	Average		4.05	4.51	23.14	35.36

**TABLE 3:** The average prices of different types of drug dispensing charges in PIVAS of Central region.

Regions	Provinces	PIVAS quantity/pieces	Common drug price/(yuan/bag)	Antibacterial drug price/(yuan/bag)	Hazardous drug price/(yuan/bag)	Parenteral nutrition solution price/(yuan/bag)
Central region	Anhui	56	3.27	3.35	17.15	19.72
	Hubei	40	9	9.98	52.44	90.68
	Shanxi	22	5	5	23.95	81.55
	Hunan	17	2.91	2.91	11.18	51.5
	Henan	13	2.12	2.26	29.24	81.86
	Average		4.46	4.7	26.79	65.06

**TABLE 4:** The average prices of different types of drug dispensing charges in PIVAS of Western region.

Regions	Provinces	PIVAS quantity/pieces	Common drug price/(yuan/bag)	Antibacterial drug price/(yuan/bag)	Hazardous drug price/(yuan/bag)	Parenteral nutrition solution price/(yuan/bag)
Western region	Yunnan	42	3.81	6.26	17.78	23.68
	Guangxi	15	3.33	3.36	14.67	21.89
	Qinghai	8	2	2	29	25.5
	Gansu	6			13.3	13.3
	Sichuan	5	2.9	2.9	7.4	9.4
	Chongqing	5	3	3	5.7	11.42
	Ningxia*	4	2	2	15	2、62.4
	Guizhou	3	2.5	4	17	15
	Inner Mongolia*	3			25	25、75
	Xinjiang*	3	3.33	4.33	3、6、14	3、6、92
	Average		2.86	3.48	13.99	27.54

**TABLE 5:** The average prices of different types of drug dispensing charges in PIVAS of Northeast region.

Regions	Provinces	PIVAS quantity/pieces	Common drug price/(yuan/bag)	Antibacterial drug price/(yuan/bag)	Hazardous drug price/(yuan/bag)	Parenteral nutrition solution price/(yuan/bag)
Northeast region	Heilongjiang	46	3.46	3.39	17.67	47.85
	Jilin	5	1	1	23	12
	Liaoning	1			44	
	Average		2.23	2.2	28.22	29.92

#### 3.2.4 Annual operating cost investigation of PIVAS in China

In light of the impact of the COVID-19 pandemic on medical institutions nationwide in 2020, it was determined to analyze the operating costs of 761 PIVAS units for the years 2019 and 2021. The research findings (as presented in Table 6) indicate that the average annual operating cost for the 761 PIVAS units was approximately 2.1371 million yuan in 2019 and roughly 2.0590 million yuan in 2021, with an average annual cost of 2.0981 million yuan (calculated as the mean of 2019 and 2021, same applies hereafter). Specifically, the average annual comprehensive operating cost for the 761 PIVAS units was 1.8748 million yuan (comprising 89.36% of the total cost), while the average annual allocation cost was 223,300 yuan (accounting for 10.64%).

**TABLE 6:** Survey results of PIVAS operating costs (*n* = 761).

Classification	Project	The average costs of 2019	The average costs of 2020	Annual operating cost
Comprehensive operating cost	Labor costs	160.00	151.35	155.68
	House decoration and maintenance expenses	10.00	10.00	10.00
	Equipment and instrument procurement costs	7.22	7.22	7.22
	Depreciation of building structures	5.75	5.75	5.75
	Electricity expenses	3.83	4.33	4.08
	Equipment and instrument maintenance fees	3.00	3.67	3.34
	Management expenditures	0.80	0.80	0.80
	Water expenses	0.60	0.63	0.61
Comprehensive operating cost	DISPOSABLE syringes	7.09	6.38	6.74
	Disposable intravenous nutrition infusion bags	5.74	6.00	5.87
	Medical order labels	3.23	3.00	3.12
	Disposable protective supplies	2.66	3.08	2.87
	Hygiene and disinfection products	1.25	1.20	1.22
	Medical waste disposal fees	1.04	1.00	1.02
	Office supplies, and other items	1.00	0.99	0.99
	Finished infusion delivery packaging bags	0.50	0.50	0.50
Total operating cost		213.71	205.90	209.81

Among the comprehensive operating costs of PIVAS, labor costs represent the highest average annual expenditure, amounting to 1.5568 million yuan annually and accounting for 74.20% of the total operating cost. The second-highest expense is related to house decoration and maintenance, averaging 100,000 yuan per year (4.77% of the total). The third-largest expense pertains to the acquisition of instruments and equipment, averaging 72,200 yuan annually (3.44%). Subsequent expenses include, in descending order, depreciation of building structures, electricity costs, maintenance of equipment and instruments, management fees, and water costs.

Among the dispensing costs of PIVAS, the highest expenditure is for disposable syringes, averaging 67,400 yuan annually, which accounts for 3.21% of total operating costs. The second-highest cost is for disposable intravenous nutrition infusion bags at 58,700 yuan per year (2.80% of total costs). The third-largest expense is for medical order labels, averaging 31,200 yuan annually (1.49% of total costs). Other notable expenses include disposable protective equipment, hygiene and disinfection products, medical waste disposal fees, office supplies, and packaging bags for finished intravenous infusion delivery, listed in descending order of cost.

#### 3.2.5 Research on fee suggestions for PIVAS in China

The research findings regarding the charging recommendations for 761 PIVAS centers indicate that there is a relatively high level of recognition for charging infusion preparation fees. Specifically, 91.06% of PIVAS centers expressed strong agreement, while 6.16% showed moderate agreement. These results suggest that, within the current medical context, the issue of PIVAS charging has garnered significant attention and acknowledgment.

Regarding whether the dispensing fees of PIVAS should be categorized into different tiers based on hospital levels, the approval rate among tertiary hospitals (58.87%) was notably higher than that among secondary hospitals (36.56%). Conversely, the disapproval rate in secondary hospitals (63.44%) exceeded that in tertiary hospitals. This discrepancy may be attributable to variations in resource allocation and service capabilities across hospital tiers.

For the charging standards of various types of drugs, the current PIVAS charging standards are universally lower than the recommended standards, as illustrated in Table 7. This suggests that when establishing charging standards, it is essential to comprehensively consider factors such as drug categories, dispensing complexity, and service quality to ensure the reasonableness and equity of the charging standards.

**TABLE 7:** Current dispensing charge standards and suggested average prices for various types of drugs (yuan per bag).

Classification	Current average charging standard	Suggested average charging standard	Difference
The price of common drugs	4.39	6.71	−2.32
The price of antibacterial drugs	5.01	9.63	−4.62
Harm caused by drug pricing	23.17	38.35	−15.18
The price of parenteral nutrition solutions	38.75	44.03	−5.28

## 4 Discussion

### 4.1 Establish a standardized and unified national PIVAS examination and evaluation criterion

The findings of this survey indicate that the departments or institutions responsible for inspecting and assessing PIVAS charges, as well as the assessment criteria, have yet to be standardized across different regions. Additionally, some PIVAS charges remain uninspected and unevaluated by any official entity. Consequently, there is an urgent need to establish a unified national PIVAS examination and evaluation standard to further advance the standardization, normalization, and homogenization of PIVAS operations nationwide.

The “Guidelines for the Construction and Management of Intravenous Medication Dispensing Centers (Trial)” issued by the National Health Commission explicitly stipulates that provincial health and wellness administrative departments should establish specialized groups under provincial pharmaceutical affairs management and pharmacotherapy committees to oversee centralized intravenous medication dispensing. These groups are tasked with providing professional technical guidance for the establishment and administration of PIVAS in medical institutions within their jurisdictions.

Consequently, the author recommends that the specialized group responsible for the centralized preparation and management of intravenous medications be designated as the primary authority for the inspection and evaluation of PIVAS. This body should conduct a thorough and systematic assessment of PIVAS from a professional technical standpoint.

In order to further promote the standardization, normalization, and homogenization of PIVAS operations, it is essential not only to identify the primary institutions responsible for inspection and evaluation but also to establish a comprehensive and scientifically rigorous evaluation index system. This system should encompass all critical aspects of PIVAS, including facility configuration, personnel training, drug management, operational standards, and quality control, ensuring that each indicator accurately reflects the actual performance of PIVAS operations: (1) Facility configuration serves as the foundation of PIVAS operations. The evaluation index system must clearly specify requirements for site layout, equipment configuration, environmental control, and other factors to ensure compliance with fundamental conditions such as sterility, dust-free environments, and temperature stability, thereby providing a secure and reliable setting for drug dispensing. (2) Personnel training represents a cornerstone of PIVAS effectiveness. The evaluation index system should mandate systematic professional knowledge and skills training for PIVAS staff, along with regular assessments, to confirm their competence and suitability for PIVAS-related tasks (Yang et al., 2023; Chen et al., 2021; Ni et al., 2021). (3) Drug management constitutes another vital component of PIVAS operations. The evaluation index system must enforce stringent oversight across all stages of drug procurement, acceptance, storage, dispensing, and usage to safeguard drug quality and safety. (4) Regarding operational standards, the evaluation index system should explicitly define all procedural norms for PIVAS activities, requiring staff adherence to minimize human-induced variability in drug quality. (5) Quality control constitutes the core of the work in PIVAS. The evaluation index system should mandate that PIVAS establish a comprehensive quality control system, carry out real-time monitoring and evaluation of every aspect of drug dispensing, and ensure that the quality and safety of drugs are effectively controlled.

By constructing this complete and scientific evaluation index system, we can conduct a comprehensive and systematic evaluation of PIVAS, rectify problems in a timely manner, and promote the continuous improvement and development of PIVAS operations. Meanwhile, this will also offer a unified guiding standard for the construction and operation management of PIVAS in various regions, facilitating the standardization, normalization, and homogenization of PIVAS work.

### 4.2 Formulate a unified national charging policy for PIVAS

When formulating a unified national charging policy for PIVAS, the relevant departments should take into consideration various factors: (1) It should be based on the complexity and riskiness of drug compounding. Although the compounding of common drugs is fundamental, it still requires rigorous process control to ensure medication safety. The compounding of hazardous drugs, due to the involvement of highly toxic and risky drugs, demands a higher technical proficiency and stricter management measures, and accordingly, the charging standard should be higher. (2) The charging standard also reflects the regional economic development level and the status of medical resources. In regions with better economic development where medical resources are abundant and medical technology is advanced, the construction and management level of PIVAS is relatively high, thus the charging standard is correspondingly higher. In some economically less developed regions, where medical resources are relatively scarce and the construction and management level of PIVAS is relatively low, the charging standard is relatively lower. Therefore, differentiated charging policies for PIVAS should be formulated for regions with different development levels or different levels of medical institutions (Nie, 2022). (3) Policy orientation and social equity need to be considered. Some regions, when formulating the charging standard, fully take into account the economic burden of patients and the reasonable control of medical expenses. By formulating a reasonable charging standard, it is ensured that patients can obtain high-quality and low-cost medical services (Yang et al., 2020). It is also necessary to enhance communication and coordination with the medical insurance department and the price control department to guarantee the compliance and operability of the charging standard (Gong et al., 2012; Lin, 2010).

## 5 Conclusion

As medical technology keeps advancing and medical reform strategies are implemented step by step, the role of PIVAS in the medical system is gradually expanding, and its effect on enhancing the quality of medical services is becoming increasingly prominent.

PIVAS operates within a Class 10,000 cleanroom environment and employs professional equipment such as biological safety cabinets. It incorporates standardized operating procedures, including a pharmacist double-check system and barcode-based traceability management, to establish a comprehensive quality control system throughout the entire workflow. By implementing a closed-loop service model—“precision dispensing–time-segmented targeted delivery”—the system not only enhances occupational safety but also effectively prevents the transmission of healthcare-associated infections. Clinically, this system significantly improves the accuracy and safety of medication therapy, providing robust technical support and management assurance for optimizing patient outcomes and ensuring high-quality healthcare services. In addition, the centralized management model implemented by PIVAS has effectively addressed the efficiency limitations inherent in the traditional intravenous medication dispensing process.

First, by leveraging an advanced information management system, PIVAS consolidates medication requests from various clinical departments in a centralized manner. Through the establishment of a closed-loop management framework that spans from drug application and dispensing to logistics distribution, the model significantly reduces the time burden traditionally associated with clinical nurses traveling to the pharmacy to collect and prepare medications independently.

Second, PIVAS facilitates a scientific separation between intravenous medication dispensing and clinical nursing duties, thereby enabling a more efficient reallocation of nursing human resources. This structural adjustment allows nurses to focus on core responsibilities such as monitoring patient conditions, conducting professional nursing assessments, and delivering health education. Consequently, the nursing service model transitions effectively from a “basic operation-oriented” approach to a “professional service-oriented” paradigm, supporting hospitals in achieving refined operation management and comprehensively enhancing the overall efficiency of the healthcare service system.

In light of this development trend, the formulation of the charging policy for PIVAS dispensing fees is an issue that demands prompt resolution. On the one hand, with the continuous progress of medical technology, the construction and management level of PIVAS will keep rising, and the establishment of the charging standard for dispensing fees requires greater scientificity and precision. The complexity and risk of drug dispensing, as well as factors such as the regional economic development level and the status of medical resources, should be fully considered to formulate a more reasonable and impartial charging standard. On the other hand, as medical reform progresses in depth, the continuous improvement of the medical security system and the reasonable control of medical expenses will become significant policy orientations. The formulation of the charging standard for PIVAS dispensing fees also needs to attach greater importance to social equity and the economic burden of patients. It is necessary to establish a reasonable charging standard to ensure that patients can access high-quality and cost-effective medical services, while safeguarding the legitimate rights and interests of medical personnel.

The costs of medical services can be determined through the use of the Resource-Based Relative Value Scale (RBRVS), and a dynamic adjustment mechanism—updated every two to three years—can be implemented to ensure a balanced approach to service valuation and cost management. This “unified benchmark with regional customization” framework offers a critical reference for China in establishing a nationally consistent medical service pricing standard. Its closed-loop process encompassing cost accounting, negotiation and consultation, and periodic updates effectively addresses the current challenges of fragmented regional disparities and delayed revisions in medical service pricing.

In summary, the formulation of the charging standard for PIVAS dispensing fees will be a process that takes into account multiple factors comprehensively and undergoes continuous improvement and optimization. It is necessary for each region to enhance supervision and evaluation to guarantee the rationality and fairness of the charging standard, and at the same time, strengthen the continuous improvement and quality control of medical services to provide patients with more superior, efficient, and safe medical services.

## Data Availability

The original contributions presented in the study are included in the article/supplementary material, further inquiries can be directed to the corresponding authors.
